# A Theory of Guilt Appeals: A Review Showing the Importance of Investigating Cognitive Processes as Mediators between Emotion and Behavior

**DOI:** 10.3390/bs9120117

**Published:** 2019-11-20

**Authors:** Aurélien Graton, Melody Mailliez

**Affiliations:** 1 LIP/PC2S, Université Savoie Mont-Blanc and Université Grenoble Alpes, 38000 Grenoble, France; 2 ISAE/SUPAERO, Université de Toulouse, 31055 Toulouse, France; melody.mailliez@isae-supaero.fr

**Keywords:** guilt, emotions, attention, persuasion, cognitive processes, decision making

## Abstract

Guilt appeals in the field of persuasion are quite common. However, the effectiveness of these messages is sometimes ambivalent. It is widely acknowledged that guilt leads people to engage into prosocial behaviors, but the effects of guilt can also be counter-productive (e.g., reactance-like effects). We argue that the explanations for these contradictions remain unsatisfactory and suggest that taking into account the implications of underlying cognitive—especially attentional—mechanisms would provide a better understanding of these paradoxical outcomes. This article provides a brief review of the literature on the link between guilt and pro-social behaviors and its classical interpretations. We propose a reinterpretation of this link by taking into account specific attentional processes triggered by the emotion of guilt. Attentional biases are, in our opinion, better predictors of the effectiveness of a message than the amount of emotion induced by the same message. This consideration should guide future research in the field of guilt appeals and pro-social behaviors. Implications, in terms of a broader comprehension of the emotion–behavior association in decision making processes, are discussed.

A picture depicts an African woman lying in a desert, holding a stylish handbag. A slogan states “Handbag: 32€. Food for a week: 4€”. Another advertisement shows a famished child, with the following statement “He’s starving, we’re not. It’s time to share”. These two examples taken from the 2000s illustrate how persuasion frequently relies on moral emotions and, especially, guilt appeals. Moral emotions can be depicted as emotions “linked to the interests or welfare either of society as a whole or at least of persons other than the judge or the agent” ([1], p. 853). Guilt is often seen as the most prototypical moral emotion [2], and it is repeatedly used in persuasion campaigns for its prosocial consequences. A wide body of research has focused on its definition (i.e., the origin of guilt) and behavioral consequences (i.e., restorative behaviors). However, there is a lack of knowledge regarding the processes linking guilt to prosocial actions. The objective of the present article is to shortly review the sometimes paradoxical effects of guilt on persuasion and to propose alternative explanations based on the investigation of underlying cognitive, especially attentional, processes. Although there are other theoretical frameworks to explain these effects, we limit our paper to the contribution of taking these cognitive processes into account.

## 1. Guilt and Prosocial Behaviors: From Reparation to Backlash Effects

It has been largely demonstrated that emotions play a regulatory function for society at large [3,4]. More specifically, guilt occurs when an individual considers that his or her actions have violated a personal moral norm and caused harm to others [2,5]. Guilt, therefore, includes a sense of personal responsibility, with some form of distress, towards others [5,6,7] (In German, the word “schuld” refers to both “guilt” and “debt”). Guilt could then encourage confessions, apologies, excuses [8], or inhibitions of subsequent behaviors [9]. The objective of these actions is to restore the relationship with others as it existed before the transgressive behavior [6]. However, reparative actions can have a broader scope than strict interpersonal relationships and can be part of the social life: “Prosocial behaviors” benefit individuals other than the self and even society as a whole [10]. The idea of a link between guilt and prosocial behavior is fairly old [11], but the experimental evidence for this association is quite recent. In the 1960s, studies showed that the adoption of transgressive behavior led to a desire to repair the harm caused (e.g., [12,13]). For instance, Regan, Williams, and Sparling [14] conducted an experiment in which participants could be led to believe that they had damaged an experimenter’s camera after he had asked them to take a picture. Participants then met a second accomplice experimenter whose bag was open. More participants who thought they had damaged the camera reported to the accomplice that his/her bag was open. In these studies, restorative behavior was attributed to guilt. However, guilt was not always assessed through experimental manipulation checks, nor was it always explicitly manipulated. At a broader scale, a meta-analysis showed a global effect of negative effects on pro-social behavior [15]. More recent studies have thus focused on better formalizing the specific causal relationship between guilt and pro-social restorative behavior. 

Ketelaar and Au [16] proposed two experiments in which participants were asked to play social-dilemma games. During the different game turns, it was possible to adopt a cooperative or an individualistic behavior. Participants experiencing guilt showed more cooperative behaviors than those in the control group (see also [17,18,19]). 

This causal relationship between guilt and prosocial behavior explains why this emotion is widely used in the fields of prevention or social marketing. In this type of persuasion, individuals are typically exposed to messages that both induce feelings of guilt and subsequently provide a way to reduce these feelings by adopting the proposed behavior. As an illustration, Coulter and Pinto [20] designed a study using a commercial message about children’s dental health. The message included “guilt inducing” sentences (e.g., “mothers who neglect their children’s dental hygiene have children with dental problems. It is your responsibility to ensure the oral follow-up of your children, do not let your family down!”), followed by a suggestion for parents to take care of their children’s health with the use of floss. Results showed that the level of guilt predicted the intention to buy dental floss. Following the same method, guilt has been used to promote condom purchase [21], anti-alcohol messages [22,23], pro-environmental behaviors [24,25], or to encourage charity giving [26,27]. More generally, guilt appeals are often used by companies or NGOs to promote their cause and products, as depicted at the beginning of this article. However, a closer look at the association between guilt and prosocial behaviors shows that this link is not as systematic as expected. 

Several studies questioned the link between guilt and reparation (e.g., [28]). In a series of five experiments, Cryder, Springer, and Morewedge [29] showed that the induction of guilt led to reparative actions only when the victim was present and could witness the restorative behavior. In other cases (e.g., absence of the victim), guilt did not lead to reparation. It therefore seems that guilt does not act as a universal trigger for restorative actions. Coulter and Pinto [20] suggested the existence of an “inverted U” curve describing the influence of guilt on persuasive messages: On the one hand, a “moderate” level of guilt would lead to greater support for the message. On the other hand, a high level of guilt would be likely to cause a rejection of the persuasive message. Other studies showed that guilt appeals in mass media campaign could be counterproductive [30]. These results are consistent with a meta-analysis conducted by O’Keefe [31] suggesting a non-linear relationship between the level of guilt expressed and the persuasive effect of a message: a high level of guilt appears to be associated with a low level of persuasion, while a low level of guilt is associated with a high level of persuasion. These paradoxical results echo the concept of “reactance” defined by Brehm ([32], see also [33]). This psychological defense mechanism occurs when an individual perceives his freedom of action to be threatened. An individual in a state of reactance will behave in such a way as to restore his freedom (or, at least, his sense of freedom), for example, by performing behaviors that are contrary to those required. As an illustration, Graton and colleagues [18] showed that guilt induction led to prosocial behavior when persuasion messages contained subtle reparation proposals. When the message was presented in a blatant or overly explicit way, guilt led to reactance-like behaviors (i.e., opposed to the persuasive message request) showing that guilt by itself is not sufficient to trigger altruistic behaviors. 

In summary, there is no systematic link between guilt and prosocial behaviors in persuasion. It also seems that the inconsistencies observed in the literature are not only due to the intensity of guilt but also to other potential factors (e.g., [25]. We argue that the current explanations for these discrepancies are not sufficient. 

A first explanation relies on the “unpleasant” nature of guilt. As a result of this aspect, individuals who experience guilt may experience distress and general discomfort [2]. Like other negative emotions (e.g., sadness, disgust), the goal for a person experiencing such a feeling is usually to “get out” as fast as possible [34,35,36] and to regain a positive emotional state. Prosocial actions make it possible to return to this positive emotional state by carrying out a “positive” action. However, the mere valence of guilt is not enough to explain why the associated behavioral tendency would be prosocial behavior, i.e., specific restorative behavior, and not, for example, relaxing while watching a TV show. Depending on the context, several other negative emotions can also lead to prosocial behaviors, like shame [37,38,39,40] embarrassment [41] (or regret (e.g., [42]). Conversely, other negative emotions do not lead to the behavioral tendency of reparation (e.g., fear leads, in the first place, to running away; see also [43]). 

According to the theory of “affect as information” (According to this theory, emotional feelings act as sources of information, in the same way as other stimuli in the environment; for a review see [44], guilt would also serve as an informational indicator that the behavior adopted, or soon to be adopted, is not compatible with a social norm of the person concerned (see [16]). Consequently, it is necessary to act in the direction of greater compliance with the standard concerned and to adopt appropriate restorative behavior (e.g., compliance with a persuasion campaign). In the case of social dilemma games, participants would attribute their guilt to poor choices in the game. Guilt would therefore only serve as valid information for those who have violated the moral standard, which would explain the effect of more prosocial behavior for those who feel guilty. “The affect as information” theory may therefore be relevant to explain the consideration that a standard has been breached and that positive action must be performed to restore a deteriorated relationship. Yet, while this theory can shed light on how a person introspectively realizes that reparation must take place, it does not provide an understanding of how and by what means an individual experiencing guilt will actually repair the harm caused.

The behavioral consequences of guilt have also been explained in terms of the reciprocity standard. It is a universal human principle that many social behaviors and interpersonal relationships could be based on the expected reciprocity of behaviors. This rule has been formalized in psychology in different ways, particularly under the name of “reciprocity standard” [45]. Many studies have considered guilt and the desire not to experience this emotion (aversion to guilt) as a consequence of the norm of reciprocity (e.g., [46],). Within this theoretical framework, moral emotions and guilt, in particular, would serve as a warning that the reciprocity standard has been violated, i.e., in the statement “I wouldn’t want others to harm me, so I should not harm them”, anticipated guilt acts as a “mediator” between moral transgression and behavior, which is close to the “affect as information” theories mentioned above. Taking into account this norm of reciprocity may help to understand the occurrence of guilt, but not the processes by which this emotion leads to reparation, especially since this link is not systematic. Other theories, like cognitive dissonance (see [47]), have been used to explain the effects of guilt in the consumer decision-making process (e.g., [48]). However, they do not take into account the way guilt could interact with other factors and, in particular, they are insufficient to interpret its sometimes paradoxical consequences (e.g., “backfire” or reactance effects). Finally, it has also been argued that these paradoxical effects could be explained by the very nature of guilt. Some authors have addressed the explanation that guilt is not an emotion, but a mere cognitive assessment of causing harm ([49], see also [50]). Following this assessment, diverse emotions could emerge, e.g., sadness or empathy over causing harm or fear of punishment or shame because of external views of the transgression. These emotions would, in turn, trigger different kinds of behavior and explain the non-linear effects of guilt. In our opinion, taking into account attentional processes could make it possible to better understand this “cocktail of emotions” potentially triggered by the emotion, or concept, of guilt. Depending on the context, guilt mixed with too much “shame” would, for instance, direct attention to the self and not toward reparation possibilities. In other cases, prototypical guilt would lead to attention being paid to the means provided for repair. One way to deepen the understanding of guilt’s role in persuasion is, therefore, in our view, to take cognitive mechanisms into account chronologically prior to behavior. 

## 2. What Cognitive Processes Are Involved in Guilt Appeals?

The shift from emotion to behavior involves physiological and cognitive intermediate processes. For example, fear is often considered to indicate a danger to the individual and leads to a state of readiness to flee by directing the attention of the person in danger both to the origin of the danger (e.g., a snake) and to escape possibilities, such as a hideout (see [51]). Under this perspective, a large body of research has examined the role of attentional mechanisms associated with emotions (e.g., [52,53,54,55,56]). Other studies have shown that emotions make information relevant to the achievement of a behavior being more “accessible” (e.g., [57,58]). Within this context, the “feeling is for doing” approach (see [43]) argues for a need to be specific when investigating the impact of emotions on decision making. However, the authors also pointed out that the link between emotion and behavior is not enough to understand the specificity of an emotion and that “it is time to move beyond the mere documentation of behavioral results of emotions to direct tests of the proposed mechanism underlying these effects. Although many studies demonstrate congruence between observed decisional effects and emotional goals, this does not conclusively attest for the idea that goal activation as a result of emotional states causes these effects.” ([59], p. 24). In fact, little work has been done to understand the cognitive mechanisms at play in the guilt–prosocial behavior relationship. A better understanding of these processes could also provide a better understanding of the global nature of the relationship between emotions and behavior. 

It has been shown that different types of cognitive processing can mediate the emotion–behavior relationship. As highlighted by the “feeling is for doing” approach, the allocation of specific attention modified by the emotional state might represent a relevant candidate to explain the mediation of the emotion–behavior relationship. In other words, attention involves the exclusion or abstraction of certain objects in order to treat others more effectively [60]. Attention is thus a corollary of the inability to process, visually or cognitively, all the information available in one’s environment [61] at one specific moment. Our objective here is not to detail the different components and specificities of attention (for a review, see, for instance, [62]) but to highlight how selective attention can account for paradoxical effects of guilt appeals. 

Many studies have highlighted the existence of attentional biases towards negative stimuli or those associated with the notion of threat for people experiencing anxiety [52,53,56]. For example, Mogg and Bradley [53] used an attentional probe task to show that anxiety disorders led to more attention being directed to negative stimuli than depression. These effects may be explained by the need to detect stimuli perceived as threatening more quickly in order to get prepared for fast action (escape). These results were replicated with other tasks, such as the “emotional Stroop test [63]. The literature on attention and anxiety shows a greater allocation of attention to the source of the anxiety feeling. In the context of an action-oriented conception of emotion, it is conceivable that the emotional state is also directed towards the means at disposal to prepare this action (e.g., the possibility to escape for fear). To date, few studies have examined the attention mechanisms specific to social and more complex emotions, such as guilt. Graton and Ric [64] showed that guilt increased attention towards items related to reparation, but this did not imply persuasion messages or real means provided to repair. This attentional selectivity towards the means of performing the action associated with the emotional state may allow for a more accurate interpretation of the behavioral effects of guilt. 

As seen above, most studies relying on guilt appeals use fairly similar methodologies: The first part of a message seeks to make the target feel guilty (via a sufficiently strong image or slogan). The second part of the same persuasion message makes it possible to engage in pro-social behavior (donation, involvement in health behavior, etc.) and thus reduce feelings of guilt by “repairing”. The problem with this type of message is that it is difficult to anticipate the risks of backlash and reactance. Meta-analyses showing inverted U-type curves (e.g., [31]) indicate a “threshold of guilt” at which too much emotional feeling causes reactance. However, they do not show the process by which this emotional “saturation” occurs, leading to a large number of persuasive messages that are relatively disappointing in their effects, even though the message intends to induce “little” guilt (see [65]). Another way to address these threshold effects is to take attention into account. Social marketing measurement tools have long been interested in these attentional processes (see [66]), particularly in the area of health and nutrition messages [67]. For example, studies have used eye-tracking techniques (for a review of these techniques, see [68]) to highlight the attentional processes involved in exposure to anti-tobacco campaigns for adolescents [69]. Wedel and Pieters [70] also showed that attention to a prevention message was increased when the number of words in the message was reduced. Oddly enough, attention measurement techniques are rarely used to study the relationships between the emotional state induced by a message and adherence to it. We support the idea that a process such as reactance is not the mere result of “too much guilt”, as can be explained in the current literature, but the result of an interaction between attentional processes activated by the emotional state and the possibility of reparation offered by the message (see, for example, [18]). In other words, it seems more relevant to us to measure the “amount” of attention allocated to a message caused by an emotion rather than the amount of emotion induced by the same message. From this attentional process could come a feeling of cognitive saturation that could lead to rejection of the message (i.e., reactance). 

## 3. Reinterpretation of Studies on Guilt and Prosocial Behavior

An approach based on emotionally triggered cognitive processes has two consequences: On the one hand, it renders possible a reinterpretation of studies investigating the link between guilt and pro-social behavior. On the other hand, and in a more theoretical way, it also calls the concept of “moral emotion” attached to guilt into question. 

In this way, a closer look at the Ketelaar and Au [16] studies shows that guilt can be induced via two different methods. In a first experiment, the source of the emotion had a direct link with the possibilities of reparation proposed afterwards (i.e., “integral”, or “endogenous”, emotion). In this case, the participants were led to feel guilty towards a partner during a cooperative game. The second part of the experiment allowed them to “repair” the harm caused by favoring this partner. In a second study, guilt was “incidental”, or “exogenous”, i.e., guilt whose origin was unrelated to the subsequent prosocial behaviors. They then participated in a social dilemma game. In both cases, guilt did cause restorative behavior. If guilt was strictly aimed at restoring the relationship degraded by the harm caused, only “integral” guilt should have caused reparation. Reparation options following “incidental” guilt should not lead to restorative or prosocial behavior, since they do not directly serve to help the victim. The results of Ketelaar & Au [16] are consistent with the “feeling is for doing” approach and a broader conception of guilt directed towards the action of repairing. In this context of a general orientation towards reparation, guilt may have served as a “cognitive trigger” by directing the attention of “guilty” participants to the possibilities of reparation offered to them. It should be noted that reparation was lacking when participants were identified as “prosocial” by nature, as opposed to “selfish” participants, the only ones to whom guilt influenced behavioral choices. This difference is also consistent with a concept of guilt that directs attention towards reparation; this attentional selectivity caused by guilt would be necessary only for participants who are not, by nature, altruistic. In contrast, it is useless for individuals who are normally oriented towards altruism and cooperation. Even more interesting is the analysis of studies showing a deficiency in the guilt–prosocial behavior association. De Hooge et al. [28] asked participants to allocate a sum of money for a birthday gift (Experiment 1) to several other individuals. Depending on the experimental conditions, participants could have been led to feel guilty towards one of these persons. In these cases, the aggrieved party obtained a larger gift, while third parties were penalized. The idea of a systematic and linear relationship between guilt and restorative behavior, even for “incidental” guilt, is not consistent with these results, in which case, third parties should also have received a larger gift, which was possible in the experimental situation. It is, however, plausible that guilt acted as a cognitive guide to direct the participants’ attention here to the origin/source of the guilty feeling. This focus on priority reparation (i.e., the injured person) could then be at the expense of other individuals. 

This approach based on attention allows a better understanding of the paradoxical effects (like inverted U curve) of guilt appeals. Cotte, Coulter, and Moore [71] showed that a strong induction of guilt led to increased awareness of the manipulative intentions of the author of an advertising message, which, in turn, led to reactance and the absence of pro-social behavior. As previously stated, an interpretation based on a traditional definition of guilt (i.e., harm caused leading to a willingness to repair) could not explain these results. Alternatively, they are compatible with the idea that guilt would trigger greater acuity in the direction of restorative opportunities in participants’ environment. As such, the manipulative intent of a message would itself become more obvious, and the message would become less effective. Similarly, Graton et al. [18] showed that, in resource-dilemma games, guilt interacted with the way reparation opportunities were presented. When these possibilities were subtly presented, guilt led to restorative behavior. On the other hand, explicit instructions (e.g., “it is necessary to mobilize to save the planet!”) provoked backlash behaviors. Again, a strict “feeling is for doing” reading of guilt cannot be sufficient to account for the interaction between emotion and type of instructions. According to this functionalist approach of emotions, a main effect of guilt should have been obtained as well, resulting from a previously activated amount of guilt. In a more subtle manner, guilt probably acted here as a “cognitive facilitator”, directing participants’ attention to opportunities for repair. When these possibilities were already prominent, it is possible that the person experiencing guilt may have felt “constrained” in his or her choices. This feeling of deprivation of liberty is characteristic of the reactance and “backlash” effects observed in all these experiments, as well as those frequently mentioned as illustrating the paradoxical effects of guilt in the field of persuasion (see [72]). The interaction of attention to repair opportunities and overly explicit proposals may have caused this impression of “forced choice” and reduced flexibility. We therefore believe that future research into studying the link between guilt appeals and persuasion will have to rely more often on attention measurement methods such as eye-tracking. 

There are, however, circumstances where traditional definitions of guilt are not relevant and the origin of guilt does not lie in the violation of a personal norm. For instance, guilt can be felt without actual transgression of norms and without having caused harm to others. These situations are described in the case of vicarious guilt [73]. In these cases, guilt is felt indirectly and results from acts and transgressions committed by others. In other contexts, the behavioral response resulting from guilt is automatically triggered in response to learned behaviors, which may range from reaction to one’s mother’s disapproving expression over one’s behavior to other scripted and automatic behaviors. These situations have been summarized in several emotional models such as the “compass of shame” model ([74], see also [75]). This model describes, for instance, how shame-guilt may lead to some sort of self-directed anger that could, in turn, lead to paradoxical effects. In these examples, attention processes can nevertheless be present as mediating processes, even if the behavioral responses are more automatic and scripted.”

## 4. Extension to Other Emotions: Mediators of the Emotion–Behavior Relationship

Taking cognitive processes into account as a mediator seems to be a way to better understand the emotion–behavior relationship. In the “feeling is for doing” approach, the transition from emotion to action through intermediate processes, such as attention, remains poorly studied. For example, attentional biases towards aggressive faces have been found for angry people compared to a control group [76] but little research has directly examined an anger-attentional bias towards stimuli related to the attack. This attentional bias towards objects related to aggressiveness (e.g., weapons, verbs such as “attack”, “strike”) could be an intermediate step between anger and aggressive behavioral tendencies. Similarly, emotions like disgust have mainly been studied in terms of increased attention to the source of this emotion (e.g., difficulty to disengage confronting a disgusting face, see [77]). It is, however, imaginable that some behaviors frequently associated with disgust (e.g., object rejection) may be preceded by attentional processes directed at “how” to achieve this behavior (e.g., stimuli representing a “garbage can” or “sink” to get rid of the object causing disgust, see [78]). Here again, “attentional probe” or eye-tracking experiments could highlight a possible attentional bias mediating the relationship between emotion and behavior. In accordance with the “feeling is for doing” theory, studies must now be carried out to better understand the nature of the association between emotion and behavior. We think that a large number of emotionally associated behaviors can potentially be explained by attentional biases directed at objects facilitating the motivated behavior. From this evidence may emerge a better understanding of the emotion–behavior relationship and a more precise use of emotional appeals in social marketing. 

## 5. Conclusions

It has been well established that emotions act as preparation for action (e.g., “feeling is for doing”, see [43]). This emotional–behavioral link is frequently studied in the literature, but the mechanisms underlying it remain poorly understood. As highlighted in the present paper, research has experimentally demonstrated the link between guilt and prosocial behaviors [16,17,19,29], leading to a large number of messages using guilt as a method of persuasion. However, reducing guilt to an emotion systematically leading to restorative actions does not theoretically allow a series of studies showing the imperfection of this guilt–restorative association to be taken into account, especially in the field of persuasion [20,71,79]. We propose the hypothesis that prosocial behavioral tendencies will go along with attentional biases. These biases may, depending on the case, facilitate prosocial behavior or, on the other hand, cause reactance. This consideration of the underlying cognitive processes of attention not only allows for a better interpretation of otherwise ambiguous results (e.g., inverted U-shaped curves, [72]) but also opens new research possibilities. It is the entire chain—*source of emotion–cognitive processes–prosocial behavior*—that must be taken into account in future research to understand the real role of emotions in fields like persuasion or health communication.
